# Implications of Adipose Tissue Content for Changes in Serum Levels of Exercise-Induced Adipokines: A Quasi-Experimental Study

**DOI:** 10.3390/ijerph19148782

**Published:** 2022-07-19

**Authors:** Kinga Humińska-Lisowska, Jan Mieszkowski, Andrzej Kochanowicz, Aleksandra Bojarczuk, Bartłomiej Niespodziński, Paulina Brzezińska, Błażej Stankiewicz, Monika Michałowska-Sawczyn, Anna Grzywacz, Miroslav Petr, Paweł Cięszczyk

**Affiliations:** 1 Faculty of Physical Education, Gdansk University of Physical Education and Sport, 80-336 Gdansk, Poland; andrzejkochanowicz@o2.pl (A.K.); aleksandra.bojarczuk@awf.gda.pl (A.B.); paulina.brzezinska@awf.gda.pl (P.B.); monika.michalowska-sawczyn@awf.gda.pl (M.M.-S.); pawel.cieszczyk@awf.gda.pl (P.C.); 2 Faculty of Physical Education and Sport, Charles University, 162-52 Prague, Czech Republic; petr@ftvs.cuni.cz; 3 Institute of Physical Education, Kazimierz Wielki University, 85-064 Bydgoszcz, Poland; barnie@ukw.edu.pl (B.N.); blasta@ukw.edu.pl (B.S.); 4 Independent Laboratory of Health Promotion, Pomeranian Medical University in Szczecin, 70-204 Szczecin, Poland; grzywacz.anna.m@gmail.com

**Keywords:** endurance exercise, anaerobic exercise, adipose tissue, IL-6, resistin, leptin

## Abstract

Human adipocytes release multiple adipokines into the bloodstream during physical activity. This affects many organs and might contribute to the induction of inflammation. In this study, we aimed to assess changes in circulating adipokine levels induced by intense aerobic and anaerobic exercise in individuals with different adipose tissue content. In the quasi-experimental study, 48 male volunteers (aged 21.78 ± 1.98 years) were assigned to groups depending on their body fat content (BF): LBF, low body fat (<8% BF, *n* = 16); MBF, moderate body fat (8–14% BF, *n* = 19); and HBF, high body fat (>14% BF, *n* = 13). The volunteers performed maximal aerobic effort (MAE) and maximal anaerobic effort (MAnE) exercises. Blood samples were collected at five timepoints: before exercise, immediately after, 2 h, 6 h, and 24 h after each exercise. The selected cytokines were analyzed: adiponectin, follistatin-like 1, interleukin 6, leptin, oncostatin M, and resistin. While the participants’ MAnE and MAE performance were similar regardless of BF, the cytokine response of the HBF group was different from that of the others. Six hours after exercise, leptin levels in the HBF group increased by 35%. Further, immediately after MAnE, resistin levels in the HBF group also increased, by approximately 55%. The effect of different BF was not apparent for other cytokines. We conclude that the adipokine exercise response is associated with the amount of adipose tissue and is related to exercise type.

## 1. Introduction

Physical exercise is reported to avert diseases, thereby contributing to human health. It is also crucially involved in metabolism. The adipose tissue is an energy reservoir [1]. Physical activity initiates triglyceride hydrolysis, following which free fatty acids are released into circulation to fuel up the working muscle [2]. However, the adipose tissue also has other roles and is no longer solely perceived as an energy storage reserve. Recent literature has highlighted the importance of body fat, which has been recently described as a bona fide immune and endocrine organ [3,4]. That is because the adipose tissue is the source of numerous biologically active compounds and cells [4]. According to previous research, adipocytes produce and release a wide range of signal-transmitting molecules. For instance, the hormone adiponectin [5] plays anti-diabetic, anti-inflammatory, and anti-atherogenic roles [6,7]. It thus facilitates crosstalk between the adipose tissue and other metabolism-related organs [8]. Other such hormones are leptin [9,10], which controls the nutritional intake [11] and is thereby known as the satiation hormone, and resistin [12], linked to type 2 diabetes [13]. In contrast to adiponectin, which enhances muscle glucose uptake and increases fatty acid oxidation [14], resistin maintains fasting glycemia [15]. Further, it has been reported that follistatin-like 1, a well-known promoter of skeletal muscle growth [16,17] is expressed in adipose tissue [18]. This tissue also produces pro-inflammatory oncostatin M (OSM) [19], which is thought to regulate the homeostatic state of the tissue and the immune cell balance [20,21]. There is also evidence that adipocytes secrete interleukin 6 (IL-6) [22,23,24], described as an adipocytokine. IL-6 is a pro-inflammatory cytokine involved in lipid and glucose metabolism, and body weight regulation [3].

The secretory function of the adipose tissue is well described [3,7,25,26]. However, far too little attention has been paid to exercise-induced changes in the secretion activity of the adipose tissue. Although several research groups examined the effect of different types of exercise on the circulating levels of adipose tissue-derived factors, the results are inconsistent. For instance, in some studies, plasma adiponectin levels were unchanged during acute cycling in healthy individuals [27], or after acute/moderate exercise in overweight/obese individuals [28,29]. By contrast, in another study, raised plasma adiponectin levels were reported in overweight elderly men undergoing 6 months of high-intensity resistance training, while moderate-intensity training did not have any effect [30]. Data on leptin have also been inconsistent and contradictory. For example, in one study, short-term exercise (<60 min) did not acutely affect leptin levels in healthy volunteers [31]. While a decrease in plasma leptin in men after a graded treadmill exercise tolerance test was shown [31], an increase in leptin levels during 41 min of cycling at 50% of maximal oxygen consumption (VO_2max_) was recorded after administration of a standardized meal [32]. This was followed by a reduction in leptin levels during recovery time, and they increased to control values after 2 h [32].

Regarding resistin, a potential link between obesity and diabetes has been proposed [15]. Consequently, resistin is mostly studied in obese individuals. Its high blood levels are linked to poor exercise capacity [33]. In overweight men, high-intensity endurance exercise does not affect circulating resistin levels up to 48 h after the exercise [34]. Similarly, resistin mRNA levels in the adipose tissue are not affected in lean and overweight subjects [35]. By contrast, data for healthy individuals subjected to exercise are scarce.

The role of follistatin is relatively established. A recent study concluded that follistatin is released into the bloodstream following an acute bout of exercise [36]. In the study, involving young and healthy men, 3 h of cycling at 50% VO_2max_ elevated follistatin blood levels but not the follistatin mRNA levels in the muscle. Similarly, resistance training is associated with an increase in circulating follistatin levels in elderly overweight women [37]. As for OSM, VO_2max_ exercise elevates OSM serum levels in young and old men [38]. These results corroborate earlier findings in a mouse model [39].

IL-6 levels increase during exercise [40]. Nonetheless, the increase is most likely driven by the muscle [41,42] and increased IL-6 output from the adipose tissue has not been convincingly demonstrated to date. The adipose tissue does not seem to contribute to the elevated arterial IL-6 levels observed during a moderate short-duration workout [43]. However, according to some authors, almost 30% of IL-6 present in the blood is derived from the adipose tissue [7].

Collectively, the above studies outline the critical role of adipocytes and the influence of physical activity on the secretory profile of the adipose tissue. It is also apparent that discrepancies exist in the data regarding adipose tissue-derived factors. In addition, side-by-side comparisons of the secretory activity of the adipose tissue in the context of body fat percentage are scarce. Further, the impact of aerobic [44] and anaerobic exercise [45] on the secretory activity of the adipose tissue is not yet clear. Little quantitative analysis of systemic response to physical activity is available. Finally, much uncertainty still surrounds the relationship between the type of exercise and adipose tissue secretion.

Accordingly, the present study was designed to determine the effect of intensive aerobic and anaerobic exercise on the serum levels of adipokines and selected cytokines considering the adipose tissue content of healthy physically active young adults.

## 2. Materials and Methods

### 2.1. Experimental Overview

Healthy and physically active male volunteers were assigned to three groups depending on the body fat content determined using a bioelectrical impedance analyzer. Body fat content was categorized as low body fat (<8%), moderate body fat (8–14%), or high body fat (>14%). These reference points were set to correspond to BMI values below 18.5 kg/m^2^ and two equal ranges within 18.5–25 kg/m^2^ [46]. Based on the WHO criteria [47], the authors of [46] calculated that approximately 8% of body fat corresponds to the BMI of 18.5 kg/m^2^ (the threshold for underweight) and approximately 20% of body fat corresponds to the BMI of 25 kg/m^2^ (the threshold for overweight). The range of 18.5–25 kg/m^2^ and, in this study, 8–20% body fat percentage range was wide and represented typical nutritional status. Therefore, it was decided to investigate participants that were closer to the underweight values and those closer to the overweight threshold. Thus, the body fat range was arithmetically divided into half, acknowledging that both size and amount of adipose tissue are correlated with adipokines secretion ([48] and [49], respectively).

The study was based on a quasi-experimental, repeated-measures design and was adapted from our previous procedure [50]. The study protocol involved two maximal tests: an anaerobic test (maximal anaerobic effort, MAnE; a double Wingate anaerobic test, WAnT) and an anaerobic test (maximal aerobic effort, MAE; Bruce treadmill test). Venous blood samples were taken at the following timepoints: immediately before, immediately after, 2 h, 6 h, and 24 h after each type of maximal physical exercise. Medical examination, the subject’s age, body composition, and height were analyzed at the study enrollment. All of the volunteers were examined by a professional physician before and after every test. Performance tests commenced with MAnE, and MAE was performed 14 days later. All of the laboratory analyses were performed at the Gdansk University of Physical Education (Gdansk, Poland).

### 2.2. Participants

Forty-eight male volunteers (21.78 ± 1.98 years old) participated in the study. The participants were assigned to three groups based on a bioimpedance body composition analysis (InBody 720, South Korea, Seoul): low body fat group (LBF, n = 16; 20.66 ± 1.91 years), moderate body fat group (MBF, n = 19; 19.86 ± 0.88 years), and high body fat group (HBF, n = 13; 20.53 ± 1.40 years). The characteristics of the groups are presented in Table 1. Recruitment to the research project was carried out based on letters of intent among the population of male students at Gdansk University of Physical Education and Sport.

The participants were physically active healthy Gdansk university students without any structured or professional sports training. All of the participants filled in the Global Physical Activity Questionnaire (GPAQ), excluding professional athletes, extremely physically active individuals, and those who were completely inactive. During the examination, 11 people were excluded because they did not meet the study’s physical activity demands. All of the participants had similar levels of physical effort exposure due to their daily schedules related to their course of study. None had a history of known diseases or reported any intake of medication due to illnesses 6 months before the study.

The participants’ description is consistent with our previous specifications published by Humińska et al. [50]. In the presented study, the population that was not qualified to participate in this project (according to the GPAQ declaration of intensive physical training) was qualified for the previously presented research. The men were representatives of the control study described in [50]. However, some of the participants involved in the current study were rejected from our previous study [50] due to the higher body fat percentage. While more physically active participants were recruited for the earlier study, the less active ones engaged in our current study. Nonetheless, all of the participants in both studies were recruited at a similar time and underwent similar testing procedures, and were treated similarly, e.g., concerning completing questionnaires and nutrition. For the entire duration of the study, the participants were instructed to maintain their everyday diet, and were asked to refrain from vigorous exercise and avoid caffeine and alcohol consumption during the 48 h preceding the testing date. Food was not consumed during testing and water was available ad libitum.

The study protocol was accepted by the Bioethics Committee for Clinical Research of the Regional Medical Society in Gdansk (KB-27/18) and the study was conducted according to the Declaration of Helsinki. Written consent was obtained from each study participant before the study. The recruits were also informed about the possibility of withdrawing consent at any time and for any reason. Before participation, the subjects were informed about the study procedures.

The lipid panel was assessed once before exercise testing, in venous blood collected into 5 mL tubes containing lithium heparin as an anticoagulant. Plasma was obtained after centrifugation at 3500 rpm for 15 min (according to manufacturer protocol). Total cholesterol, triglycerides, high-density lipoprotein (HDL) cholesterol, and low-density lipoprotein (LDL) cholesterol were quantified using spectrophotometric methods.

Laboratory kits (Randox Laboratory Ltd., Crumlin, UK) were used for all of the biochemical analyses and sample absorbance was read using a UV–vis spectrophotometer (DREL 3000 HACH). The results of total cholesterol, HDL, LDL, and triglyceride analyses are outlined in Table 2.

### 2.3. Measurement of Anaerobic and Aerobic Fitness Level

The subjects’ performances were assessed using the WAnT (for MAnE) and the Bruce treadmill test (for MAE). Table 3 summarizes the individuals’ fitness levels.

#### 2.3.1. Maximal Anaerobic Effort

The maximal anaerobic effort was determined using a twice repeated WAnT on a cycle ergometer (Monark 894E, PeakBike, Sweden). The procedure was described previously and adapted from Kochanowicz et al. [51]. The saddle height was adjusted for each participant (knees remaining slightly flexed after the completion of the downward stroke for the final knee angle of approximately 170–175°). All of the participants started with a standardized warm-up on the cycle ergometer (5 min at 60 rpm, 1 W/kg). During the test, each participant pedaled with maximum effort for 30 s against a fixed resistive load of 75 g/kg of total body mass, as recommended by Bar-Or [52]. After that, the participants had a 30 s break and the WAnT was repeated in the same manner, with maximum verbal encouragement.

#### 2.3.2. Maximal Aerobic Effort

For MAE, the Bruce protocol on an electric treadmill (h/p/cosmos, Germany) was implemented as described elsewhere [50]. Briefly, after a standardized warm-up, each participant undertook running with an increasing load, including velocity and treadmill inclination. During the test, the participants wore a facemask connected to a pulmonary gas exchange analyzer (Quark CPET, Cosmed, Italy). The test ended when the subject could not continue because of fatigue or other conditions.

### 2.4. Blood Sample Collection and Measurements of Selected Markers

The following procedures were adapted from our previous study [53]. Blood (9 mL) was collected five times: immediately before, immediately after, 2 h, 6 h, and 24 h after every test. Venous blood samples were collected into Sarstedt S-Monovette tubes (S-Monovette^®^ Sarstedt AG&Co, Nümbrecht, Germany) without an anticoagulant for serum separation but containing a coagulation accelerator. The serum was separated using standard laboratory procedures, aliquoted, and frozen at −80 °C until further analysis.

Levels of the following markers were determined: adiponectin, follistatin-like 1, IL-6, leptin, OSM, and resistin. The analyses were performed using a MAGPIX fluorescence detection system (Luminex Corp., Austin, TX, USA) and Luminex assays (Luminex Corp.- Luminex Human Magnetic Assay (6-Plex).

### 2.5. Statistical Analysis

The statistical procedures used were adapted from our previous work [50]. The descriptive statistics included mean ± standard deviation (SD) for all of the measured variables. One-way ANOVA was used to investigate intergroup differences in physical, lipid profile, and performance characteristics. Two-way ANOVA with repeated measures (RM: baseline and immediately after, and 2 h, 6 h, and 24 h after exercise) was used to investigate the levels of biochemical markers after MAE and MAnE depending on the participants’ percent body fat (group: LBF, MBF, and HBF). To assess differences in particular subgroups, Tukey’s post hoc test was used. In addition, the effect size was calculated by using eta-squared statistics (ƞ^2^). Values equal to or more than 0.01, 0.06, and 0.14 indicated a small, moderate, and large effect, respectively. The Shapiro–Wilk and Levene’s tests were performed to check the normal distribution and homogeneity of variance, respectively. The total sample size of 48 participants was determined using the G*Power software ver. 3.1.9.4. (Franz Faul et al., Universität Kiel, Kiel, Germany) for the moderate effect size and power of 0.95. All of the analyses were performed using Statistica 12 (StatSoft Inc., Tulsa, OK, USA). The level of significance was set at *p* ≤ 0.05.

## 3. Results

### 3.1. Maximal Anaerobic Effort

**Figure 1:**
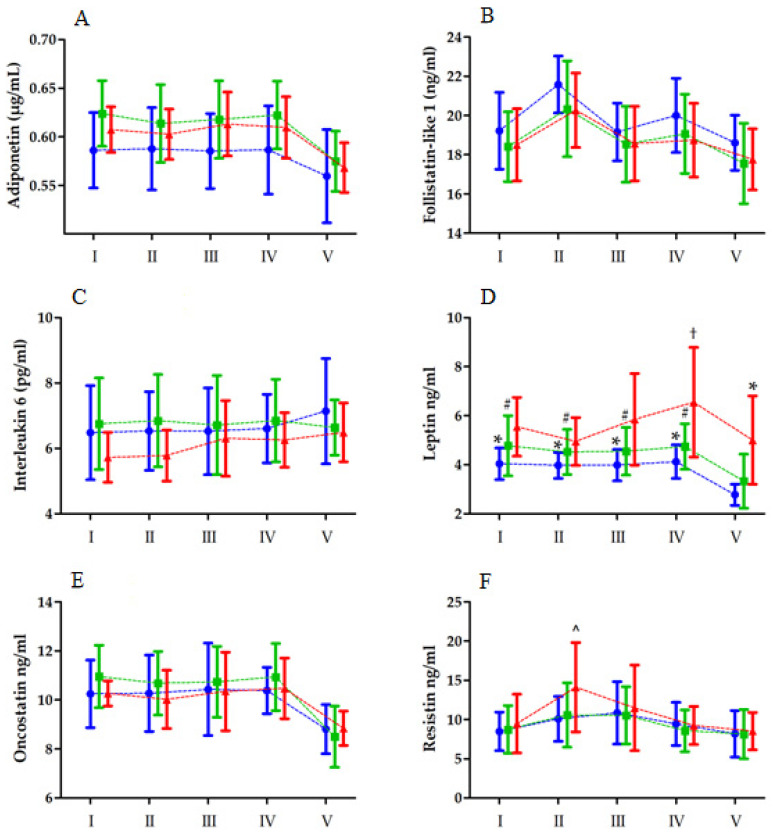
Changes in the levels of biochemical markers ((**A**)—adiponectin, (**B**)—follistatin like 1, (**C**)—interleukin 6, (**D**)—leptin, (**E**)—oncostatin, (**F**)—resistin) after the double 30 s Wingate anaerobic test (means and standard deviations are shown) in the low body fat (blue), moderate body fat (green), and high body fat (red) groups. I, baseline; II, immediately after exercise; III, 2 h after exercise; IV, 6 h after exercise; V, 24 h after exercise. Significant difference vs. * low body fat group 24 h after exercise at *p* < 0.05; # moderate body fat group 24 h after exercise at *p* < 0.05; † high body fat group immediately after exercise at *p* < 0.05. ^ Significant difference vs. high body fat group at baseline, immediately after, and 6 and 24 h after exercise at *p* < 0.05. MAnE exerts significant interactions between the group and RM factor on leptin and resistin ((D) and (F), respectively).

Changes in biochemical marker levels after MAnE are shown in Figure 1. In contrast to IL-6, the analysis of variance revealed a significant RM effect for all of the tested biochemical markers. In turn, the effect of the group factor was apparent for adiponectin and leptin. Significant interactions of the group and RM factor were noted for leptin and resistin. A post hoc analysis revealed a significant increase in serum leptin levels 6 h after exercise, compared with the values recorded immediately after exercise, only in the HBF group (by 34.35%). On the other hand, leptin levels in the LBF and MBF groups were unchanged from baseline to 6 h after exercise. Leptin levels significantly decreased in the LBF (by 31.32%) and MBF groups (30.33%) after 24 h. Despite the decrease noted 24 h after MAnE in each group, leptin levels in the HBF group were significantly higher than in the LBF group.

A post hoc analysis of serum resistin revealed a significant increase in the HBF group immediately after MAnE. Resistin levels 6 h and 24 h after exercise decreased to values comparable with those at baseline (Table 4).

### 3.2. Maximal Aerobic Effort

**Figure 2:**
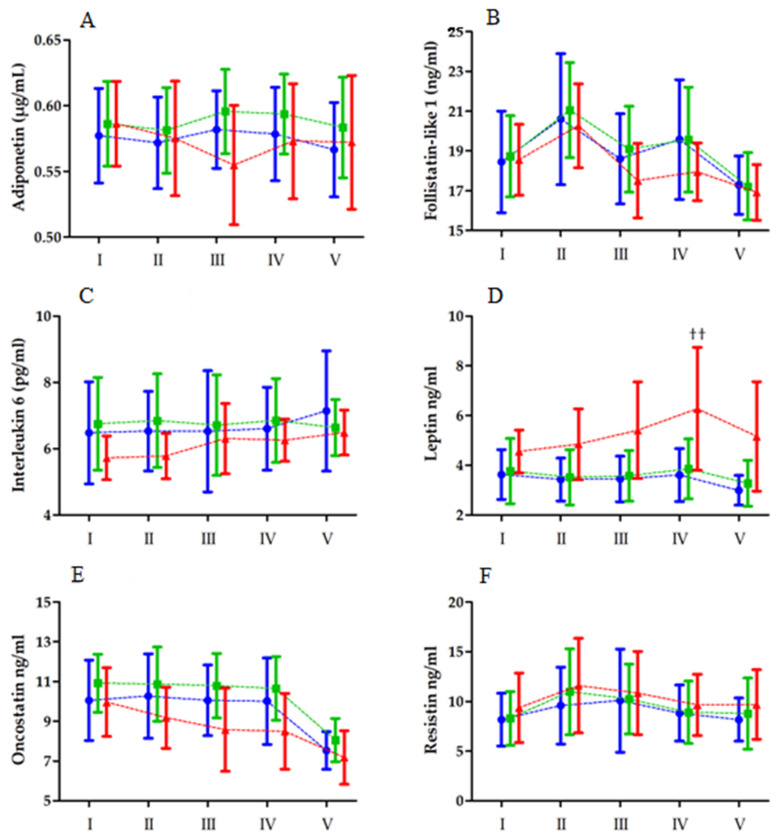
Changes in the levels of biochemical markers ((**A**)—adiponectin, (**B**)—follistatin like 1, (**C**)—interleukin 6, (**D**)—leptin, (**E**)—oncostatin, (**F**)—resistin) after the Bruce treadmill test (means and standard deviations are shown) in the low body fat (blue), moderate body fat (green), and high body fat (red) groups. I, baseline; II, immediately after exercise; III, 2 h after the exercise; IV, 6 h after exercise; V, 24 h after exercise. †† Significant difference vs. high body fat group before and immediately after exercise, vs. low and moderate body fat group 6 h after exercise. MAE exerts a significant interaction between the group and RM factor on leptin (**D**).

Changes in the levels of biochemical markers after MAE are presented in Figure 2. Similar to MAnE, the analysis of variance revealed a significant effect of the time factor on all of the tested biochemical markers, in contrast to IL-6. However, the effect of the group factor was noted for IL-6, leptin, and OSM.

The analysis of variance revealed a significant interaction of effects only for serum leptin levels (Table 5). As in the case of MAnE, serum leptin levels were highest 6 h after exercise. However, a significant increase was noted from the baseline values (35.48%) and immediately after exercise (27.29%). In addition, leptin levels 6 h after MAE in the HBF group were significantly higher than those in the LBF and MBF groups.

## 4. Discussion

In this study, we set out to determine changes in the circulating adipokine levels in association with the amount of adipose tissue and inflammation, in response to intensive aerobic and anaerobic exercise in physically active young adults. To the best of our knowledge, this is one of the first studies that mainly concentrates on the association between aerobic and anaerobic exercise and the endocrine function of the adipose tissue. A direct comparison of the results to those of other studies is therefore limited because the discussed problem is novel.

The analysis revealed that exercise-induced changes in the serum adipokine levels are associated with the amount of adipose tissue and related to the type of physical effort. For both anaerobic and aerobic exercise, leptin levels increased substantially only in the HBF group and reached a peak 6 h after exercise. Most studies investigating the effects of short-term exercise on leptin report a reduction or no changes in leptin levels [54,55,56,57,58,59,60,61]. For example, a transient decline in leptin levels (6–14%) in individual subjects, up to 120 min post exercise, was shown in men and women 18–55 years of age and with a BMI corresponding to that of the HBF group in the current study after a treadmill test following the Bruce protocol to exhaustion [31,62]. Similarly, longer exercise, i.e., 1 h of running at 50% VO_2max_, caused a transient decrease (28%) of leptin levels in obese women up to 60 min after the exercise [63]. A long-lasting physical effort was associated with a decline in leptin levels [63]. This decrease might be related to the elevated production of non-esterified fatty acids during exercise, which is inversely correlated with leptin levels [64].

Considering MAnE, one study reported no immediate effect of a single WAnT on leptin levels in moderately active men with moderate body fat (BMI = 23.78 kg/m^2^) [65]. On the other hand, Guerra et al. [66] demonstrated that leptin levels in skeletal muscle are reduced in response to a single WAnT exercise by 17% and 26%, 120 and 240 min after exercise, respectively. In another study, four repeated WAnTs decreased leptin levels by up to 20% within the first 90 min after exercise in young overweight/obese women [67]. While we did not detect any significant changes in leptin levels immediately after a double WAnT in the current study, we did observe significantly higher leptin levels 6 h after the exercise in the HBF group than those in the LBF and MBF groups. Most likely, we did not observe a significant reduction in leptin levels in the current study because the applied exercise required only half of the energy expenditure of that applied by Vardar et al. [67]. In addition, we used a different methodological approach than that in [67], comparing five timepoints instead of three. This resulted in a relatively lower sensitivity to detect small changes between analyzed parameters. In summary, the immediate and short-term effects of MAnE on serum leptin levels are probably associated with the amount of adipose tissue and the exercise volume.

The leptin data are intriguing, as the levels increased 6 h after exercise for both types of exercise only in the HBF group. Duzova et al. [68] also reported an increase in serum leptin levels following the implementation of the Bruce treadmill protocol. However, the increase occurred immediately after the exercise. This presumably could be associated with different amounts of adipose tissue (32% in ref. [68] vs. 17.4% in our study) and the different sex of the participants (females in [68] vs. males in our study). It should be noted that in the current study, we also observed the tendency to increase immediately after MAE and as soon as 2 h after MAnE. Of note, the increase reported by Duzova et al. [68] was observed only after 12 weeks of jogging–walking training. Other studies investigating changes in leptin levels after acute exercise focused on the immediate effects in untrained individuals or athletes with lean body types. Therefore, an increase in serum leptin levels might be observed only in individuals with an increased amount of adipose tissue and who are trained to withstand intense aerobic exercise. It is accepted that leptin resistance might not be a simple short-term biomarker of satiety [69] and that leptin levels are a function of body fat and food availability [70].

Besides the immediate and short-term effects of exercise, others have reported a delayed effect on leptin levels. For instance, it has been reported that the deferred (24–48 h) decline in leptin levels after exercise mainly depends on the energetic expenditure [71,72,73], so the higher the energy expenditure, the shorter the delay in leptin level decrease, even within a few hours in the case of prolonged exercise [71,72,73]. We here showed a reduction of leptin levels 24 h after only MAnE, which was more pronounced in the LBF group than in the HBF group. In the case of MAE, a tendency for leptin levels to decrease after 24 h was only apparent in the HBF group. It is possible that the rise in leptin levels described earlier compensated for the reduction observed in participants with a relatively low amount of adipose tissue.

Similar to the leptin data, the current study revealed a decrease in serum adiponectin levels only 24 h after MAnE. This was observed regardless of the amount of adipose tissue. The knowledge of the late effects of exercise on adiponectin levels is limited. Jamurtas et al. [34] reported no difference in adiponectin levels in overweight men up to 48 h after 45 min of exercise at 65% VO_2max_ intensity. A similar lack of change was documented 17–22 h after an ultramarathon run [74]. The difference in outcomes in [74] and in the current study could be related to the different types of effort and energy expenditure tested.

According to previous studies, strenuous exercise augments adiponectin levels or does not affect them at all [54,55,67,75]. Conversely, others reported a reduction in serum adiponectin levels after five repeated WAnTs in sedentary young adults [76]. The lack of immediate effect in the current study could be explained by the notion that raised catecholamine levels during intense exercise hamper adiponectin secretion [29]. Augmentation of adiponectin levels is likely related to the changes in body composition, instead of a specific manner in which an exercise is being performed [34], when energy expenditure is limited. Resting serum adiponectin levels are diminished in overweight/obese individuals [77]. While the amount of adipose tissue in the HBF group was higher than that in other groups, we did not observe any differences in adiponectin levels at rest or after exercise. The current study suggests that it is unlikely that changes in the adiponectin levels after exercise are related to the amount of adipose tissue in physically active non-obese young adults.

In the case of resistin, the effect of the amount of adipose tissue was only observed in MAnE, with resistin levels elevated immediately after exercise in the HBF group. Similarly, prolonged strenuous exercise, such as marathon running, leads to an increase in serum resistin levels among athletes [74,78,79]. Other studies investigating acute effects of exercise on serum resistin levels in overweight/obese participants reported no changes [34] or a transient decrease after 90 min [67] after four repeated WAnTs. These discrepancies could be explained by the differences in the amount of adipose tissue and physical activity of study participants (VO_2max_ of 55.28 mL/kg/min and a mean relative power output of 7.87 W/kg in the current study, with 32.8 mL/kg/min in Jamurtas et al. [34] and 3.7 W/kg in Vardar et al. [67], accordingly). Hence, the aerobic and anaerobic capability could play a role in post-exercise changes in resistin levels, especially since the changes in serum resistin levels (associated with a 10.8% mean power output increase) were no longer apparent after 19 days of high-intensity interval training [67]. The increase in resistin levels in the HBF group immediately after MAnE suggests dysregulation of adipose tissue activity. This secretory factor has been linked to insulin resistance and diabetes. Therefore, it is important to analyze it in the context of metabolic syndrome [15,80,81].

In the current study, FSTL-1 levels did not differ between groups. In both MAnE and MAE, we observed an increase in serum FSTL-1 levels immediately after exercise and a decrease 24 h after exercise, in relation to rest values. The observed increase after exercise is in accordance with the results of Mendez-Gutierrez et al. [70], Mieszkowski et al. [82], and Kon et al. [83], who showed that FSTL-1 levels increase after an endurance exercise session consisting of a maximum effort test on a treadmill, a marathon run, and four repeated WAnTs, accordingly [70]. Of note, among the three cited studies, only Mieszkowski et al. [82] investigated the late (24–48 h) response post exercise and, in contrast with the findings of the current study, no difference in comparison to the baseline was apparent. Similarly, OSM data indicated no effect of the adipose tissue on changes induced by either MAE or MAnE. Overall, we observed a reduction of serum OSM levels 24 h after both exercise types regardless of the group. Not much is known about the effect of acute exercise on OSM. According to one study, a marathon run does not substantially alter OSM levels [82]. While the amount of energy expenditure in the current study drastically differs from that in [81], it was also previously shown that a 12-week training either leads to an increase in OSM levels [84] or has no effect on them [85]. Therefore, the impact of exercise on OSM levels remains to be explored.

IL-6 is considered to be both pro- and anti-inflammatory. It has been shown that obese individuals are prone to increase circulating IL-6 levels [86]. In the current study, while no obese participants were considered, we did evaluate participants (physically active men) with a wide range of adipose tissue content. Within that range, IL-6 levels in the HBF group were not higher than those in other groups. Of note, IL-6 levels in the HBF group tended to be lower than those in the MBF group, both at rest and after exercise.

It is well known that intense physical activity leads to an increase in circulating IL-6 levels [55], especially after prolonged strenuous exercise, such as a marathon run [75]. Surprisingly, in the current study, we did not observe any changes in IL-6 levels. Similar findings, i.e., no change, were reported by Lira et al. [87] after four repeated WAnTs of either the upper or lower limbs among judo athletes. The same was observed by Williams et al. [88], who showed that just 60 min of endurance effort (65% VO_2max_) induced significant changes in IL-6 levels, while four repeated WAnTs did not. According to Lira et al. [87], the maintenance of IL-6 levels can be related to increased serum glucose after exercise sessions [89], as trained individuals are typically characterized by elevated muscle glycogen storage [90]. Furthermore, no changes after 60 min of either moderate (50% VO_2max_) or intense (70% VO_2max_) effort were observed among overweight men [29]. On the other hand, Bilski et al. [61] reported that a single WAnT procedure increases plasma IL-6 levels. Similarly, Antosiewicz et al. [91] demonstrated that three repeated WAnTs lead to a rise in IL-6 levels in both untrained and trained participants. While the effect of different effort types is unclear, it appears that the exercise-induced increase in IL-6 levels is not associated with the amount of adipose tissue in non-obese young adults.

Of note, in the current study, the anaerobic and aerobic performance was similar in all of the groups, regardless of the amount of adipose tissue. As mentioned above, this could be a factor in the case of specific findings for participants characterized by high adipose tissue content but still capable of high performance.

In the present study, the HBF population was at an increased risk of metabolic syndrome, as indicated by the amount of adipose tissue and lipid profile characteristics (mainly, increased triglycerides, cholesterol, LDL, etc., and high body fat allocation). However, they were all young individuals in their twenties and, from this point of view, it is encouraging that HBF did not manifest significant alterations in most of the tested biochemical parameters. This implies that the secretory function of the adipose tissue is somehow balanced, so that even at such a distant timepoint as 24 h after both types of exercises, no striking effects were seen. Both aerobic and anaerobic exercise are distinctly correlated with improved health. Further work is required to determine the effects of aerobic and anaerobic exercise on the endocrine function of the adipose tissue and to establish the superiority of one type of exercise over another.

It is well known that intense physical exercise generates a robust inflammatory response, characterized by a great outflow of inflammatory mediators (cytokines, exerkines, interferons, growth-regulating factors, and other peptides). It should be emphasized that these mediators also act on each other, by inhibiting or stimulating other peptides, and their complex interconnection of mutual relations underpins the term “cytokine network” [92,93].

The exercise applied in the current study was relatively brief, only lasting up to several min (time to exhaustion) for MAE and 60 s for MAnE. Many studies employing longer training programs reported the anticipated anti-inflammatory effects [94]. Hence, in the current study, one could speculate that the two types of effort did not affect the inflammation profile, as any such change was not evident, e.g., in IL-6, oncostatin M, and follistatin levels. Nonetheless, we acknowledge that these markers are pleiotropic. 

Further study could assess the short-term effects of both types of exercise on the levels of pro-inflammatory mediators, e.g., TNF-α and IL-1β, and those of anti-inflammatory mediators, e.g., TGFβ1 and IL-10. The duration of the inflammatory response varies greatly depending on the nature and duration of the stimulus. However, such data would be indicative of inflammation induced by aerobic and anaerobic exercise, if induced at all. In addition, experimental protocol enhancement, e.g., the extension of the experiment by several days in combination with dietary control and body fat measurements, could indicate whether the alterations in biochemical parameters have a disadvantageous or beneficial role in improving the anaerobic and aerobic performance of young adults with high body fat content [67].

### 4.1. Limitations

Considering the secretory endocrine activity of the adipose tissue, one should remember that the cellular response of adipocytes always depends on the number of adipose cells. In the current study, we focused on healthy physically active young adults with a BMI below 30 kg/m^2^, and with a relatively low or moderate fat percentage. Subsequent studies should also consider obese individuals with a BMI over 30 kg/m^2^ and over 35 kg/m^2^. On the other hand, it should be acknowledged that the amount of adipose tissue is not the only factor regulating its secretory activity. We have to keep in mind that the secretory activity may also be influenced by the location and variability of adipose tissue. For instance, muscle mass and the number of myocytes determine myokine activity, which affects other cells’ secretions. It is well established that adipocyte secretory activity should be assessed in relation to myocyte secretory activity as one of the main regulating factors. The age of the study population is another limitation of the current study. Our study focused on healthy young men, in whom any pathological changes related to the disturbance of endocrine activity of the adipose tissue might not be as pronounced, so that the compensatory mechanisms would hamper the progression of an unfavorable secretory trend. Further, the low number of participants and heterogeneity within groups is another limitation of the study, as that could bias the results. However, according to the sample size calculation, the number of participants involved in the current study exceeds the necessary number of participants for the selected study design. Furthermore, it has been shown that a three-group model similar to the one used in the current study and with a total sample size of 30–45 is robust even without heterogeneity of variance, up to a variance ratio of 3.0 [95]. Hence, the risk of bias in the used approach is limited.

### 4.2. Practical Application

The current manuscript complements earlier findings on the secretory/endocrine activity of the adipose tissue. The presented research has several practical applications. First, it points to the observation that differences in adipose tissue content influence many physiological factors. Of course, the exclusive focus on biochemical cellular responses is a limitation. However, an adiposity increase of only a few percent affects most of the physiological characteristics of training individuals and differentiates the exercise response. At the initial stage of training, obese individuals do not respond to acute aerobic and acute anaerobic activity, and they much better tolerate low- and moderate-intensity exercise. These types of exercises should be implemented at the initial stage of training when an individual starts performing physical activity. After a short period of adaptation to a certain type of activity, i.e., its duration and intensity, acute high-intensity exercise should be introduced. This would have a greater effect on cardiopulmonary efficiency than intense training counting from its beginning. Of course, this relationship concerns only the tissue-dependent response. Further recommendations should be carefully examined.

## 5. Conclusions

The current study revealed different responses of serum adipokine levels, depending on the amount of adipose tissue, to different types of exercise. The circulating leptin and resistin levels after an intense effort in physically active young adults with relatively high body fat were higher than those in physically active young adults with relatively low body fat.

## Figures and Tables

**Table 1:** Physical characteristics of the participants (n = 48).

Variables	Unit	LBF (*n* = 16)	MBF (*n* = 19)	HBF (*n* = 13)
		Mean ± SD	Mean ± SD	Mean ± SD
Height	cm	181.60 ± 3.85	179.23 ± 7.27	182.26 ± 7.23
Weight	kg	74.04 ± 8.03	76.20 ± 9.11	85.37 ± 11.84 *#
BMI	kg/m^2^	22.47 ± 2.58	23.89 ± 2.33	25.61 ± 2.28 *
Skeletal muscle mass	kg	39.87 ± 4.80	39.13 ± 4.66	40.12 ± 5.32
Body fat mass	kg	4.56 ± 1.21	8.51 ± 1.80 *	15.06 ± 4.74 *#
Percent body fat	%	6.15 ± 1.48	11.12 ± 1.89 *	17.46 ± 3.86 *#

**Note:** LBF, low body fat; MBF, moderate body fat; HBF, high body fat. * Significant difference vs. LBF at *p* < 0.05, # significant difference vs. MBF at *p* < 0.05.

**Table 2:** Lipid profile characteristics of the participants (n = 48).

Variables	Unit	LBF (*n* = 16)	MBF (*n* = 19)	HBF (*n* = 13)
		Mean ± SD	Mean ± SD	Mean ± SD
Total cholesterol	mg/dL	137.00 ± 20.71	156.04 ± 31.05 *	156.31 ± 21.50 *
High-density lipoprotein (HDL) cholesterol	mg/dL	49.47 ± 8.49	54.79 ± 16.13	46.75 ± 7.31
Low-density lipoprotein (LDL) cholesterol	mg/dL	75.17 ± 16.81	86.08 ± 23.14	93.75 ± 20.27 *
Cholesterol non-HDL	mg/dL	88.35 ± 18.65	98.20 ± 25.61	109.68 ± 24.48 *
Triglycerides	mg/dL	60.35 ± 17.94	65.70 ± 18.66	78.93 ± 35.53

**Note**: LBF, low body fat; MBF, moderate body fat; HBF, high body fat. * Significant differences vs. LBF at *p* < 0.05.

**Table 3:** Performance characteristics of the participants (*n* = 48).

Variables	Unit	LBF (*n* = 16)	MBF (*n* = 19)	HBF (*n* = 13)
		Mean ± SD	Mean ± SD	Mean ± SD
Maximal anaerobic effort				
Relative peak power of the 1st WAnT	W/kg	10.47 ± 0.99	10.57 ± 0.99	9.75 ± 0.94
Relative mean power of the 1st WAnT	W/kg	8.24 ± 0.72	8.40 ± 0.66	7.87 ± 0.48
Relative peak power of the 2nd WAnT	W/kg	7.58 ± 0.83	7.79 ± 0.70	7.56 ± 0.67
Relative mean power of the 2nd WAnT	W/kg	5.98 ± 0.60	5.97 ± 0.54	5.53 ± 0.54
Maximal aerobic effort				
Maximal ventilation	L/min	148.80 ± 21.83	157.41 ± 18.33	146.55 ± 26.54
Maximal oxygen uptake	ml/min/kg	57.72 ± 7.83	59.25 ± 5.17	55.28 ± 7.97
Maximal heart rate	beats/min	190.40 ± 9.74	188.25 ± 15.55	191.33 ± 9.71

**Note:** LBF, low body fat; MBF, moderate body fat; HBF, high body fat; WAnT, Wingate anaerobic test.

**Table 4:** Two-way (3 groups × 5 repeated measures) ANOVA of biochemical marker levels induced by a double 30 s Wingate anaerobic test in low, moderate, and high body fat groups.

Variable	Effect	F	df	*p*-Value	Effect Size (η^2^)	Post Hoc Outcome
Adiponectin	Group RM Group × RM	3. 85 30.64 1.24	2, 45 4, 180 8, 180	0.02 * <0.01 ** 0.28	0.14 0.40 0.05	LBF < MBF V < I, II, III, IV
Follistatin-like 1	Group RM Group × RM	1.74 39.63 0.41	2, 45 4, 180 8, 180	0.18 <0.01 ** 0.91	0.07 0.46 0.01	II > I, III, IV, V; V < I, III, IV
Interleukin 6	Group RM Group × RM	1.45 1.91 0.88	2, 45 4, 180 8, 180	0.25 0.31 0.53	0.05 0.02 0.03	
Leptin	Group RM Group × RM	7.22 21.20 2.20	2, 45 4, 180 8, 180	<0.01 ** <0.01 ** 0.02 *	0.24 0.32 0.10	LBF < HBF IV < I, II, III, IV; IV > II I-IV_LBF_ > V_LBF_ I-IV_MBF_ > V_MBF_ II_HBF_ < IV_HBF_ IV_LBF_ < IV_HBF_ V_LBF_ < V_HBF_
Oncostatin	Group RM Group × RM	0.65 34.22 1.32	2, 45 4, 180 8, 180	0.52 <0.05 * 0.24	0.02 0.43 0.05	V < I, III, IV
Resistin	Group RM Group × RM	0.66 24.18 2.56	2, 45 4, 180 8, 180	0.52 <0.01 ** 0.01 *	0.02 0.34 0.10	II > I, IV, V; V < III I_HBF_, IV_HBF_, V_HBF_ < II_HBF_

**Note:** LBF, low body fat; MBF, moderate body fat; HBF, high body fat; I, baseline; II, immediately after exercise; III, 2 h after exercise; IV, 6 h after exercise; V, 24 h after exercise. * Significant difference at *p* < 0.05, ** significant difference at *p* < 0.01.

**Table 5:** Two-way (3 groups × 5 repeated measures) ANOVA of biochemical marker levels induced by the Bruce treadmill test in low, moderate, and high body fat groups.

Variable	Effect	F	df	*p*-Value	Effect Size (η^2^)	Post Hoc Outcome
Adiponectin	Group RM Group × RM	1.12 0.77 1.82	2, 45 4, 180 8, 180	0.33 0.54 0.07	0.05 0.01 0.08	
Follistatin-like 1	Group RM Group × RM	0.54 28.17 0.84	2, 45 4, 180 8, 180	0.58 <0.01 ** 0.57	0.03 0.41 0.04	II > V > I, III, IV
Interleukin 6	Group BTT Group × RM	3.77 1.92 1.66	2, 45 4, 180 8, 180	0.04 * 0.11 0.53	0.05 0.02 0.03	MBF > HBF
Leptin	Group RM Group × RM	6.32 9.18 2.26	2, 45 4, 180 8, 180	<0.01 ** <0.01 ** 0.02 *	0.24 0.19 0.10	LBF, MBF < HBF V < III, IV; IV > II I_HBF_, II_HBF,_ IV_LBF_, IV_MBF_ < IV_HBF_
Oncostatin	Group RM Group × RM	3.36 6.62 1.25	2, 45 4, 180 8, 180	0.05 * <0.01 ** 0.27	0.13 0.14 0.05	MBF > HBF V < I, II, III, IV
Resistin	Group RM Group × RM	0.39 12.53 0.58	2, 45 4, 180 8, 180	0.67 <0.01 ** 0.78	0.02 0.23 0.10	I, IV, V < II, III

**Note:** LBF, low body fat; MBF, moderate body fat; HBF, high body fat; RM, repeated measure; I, baseline; II, immediately after exercise; III, 2h after exercise; IV, 6 h after exercise; V, 24 h after exercise. * Significant difference at *p* < 0.05, ** significant difference at *p* < 0.01.

## Data Availability

The data presented in this study are available on request from the corresponding author. The data are not publicly available due to ethical reasons.
